# Health belief model of parents’ COVID-19 vaccination intentions for children: perceived benefits and barriers in Indonesia

**DOI:** 10.3389/fpubh.2024.1485416

**Published:** 2025-01-29

**Authors:** Eka Wuri Handayani, Dyah Aryani Perwitasari, Fredrick Dermawan Purba

**Affiliations:** ^1^Department of Pharmacy, Faculty of Health Sciences, Universitas Muhammadiyah Gombong, Kebumen, Indonesia; ^2^Department of Pharmacology and Clinical Pharmacy, Faculty of Pharmacy, Universitas Ahmad Dahlan, Yogyakarta, Indonesia; ^3^Department of Psychology, Faculty of Psychology, Universitas Padjajaran, Bandung, Indonesia

**Keywords:** guidance, parents’ intention, childhood vaccination, COVID-19, qualitative study, Indonesia

## Abstract

**Introduction:**

The uptake of vaccines against COVID-19 remains low. Some barriers to childhood vaccination uptake persist, such as parents’ assumption that children are at lower risk of severe COVID-19 and tend to be asymptomatic carriers. This study aims to develop guidance for in-depth interviews for a future qualitative study based on a cross-sectional quantitative study of parents with school-age children.

**Methods:**

This study adopted a cross-sectional design. The study population comprised parents of 6–11-year-old children in the Centra Java province who had received the COVID-19 vaccine or not. The data were collected from August 2023 by filling in an online questionnaire. The sample size was calculated using formulation in OpenEpi for 95% confidence levels, with a statistical power of 80%.

**Results:**

Our study finds that perceived benefit and perceived barriers are the two domains that most significantly influenced the parents’ intention to vaccinate their children. In our study, there was no significant association between parent gender and the intention to vaccinate their children. Our study shows that parents’ acceptance of vaccinating their children is high. We emphasized questions related to benefits and barriers in the interview. The questions on perceived benefits explored the advantages of COVID-19 vaccination. The content on perceived barriers examined the concerns of parents, the information influencing their decision to vaccinate their child, the procedure vaccination and the effect after vaccination.

**Discussion:**

The significant association between parents’ intention to vaccinate their children and the perceived benefits and perceived barriers to vaccination generated guidance for in-depth interviews in the qualitative study. The health belief model should be further explored in Indonesia because of the potential external factors that may influence parents’ intention to vaccinate their children.

## 1 Introduction

The COVID-19 vaccine received emergency authorization from the Food and Drug Administration (FDA) in 2020 (1). This was followed by the approval of COVID-19 vaccines in many countries. However, in some countries, the uptake of COVID-19 vaccination has remained low because of core beliefs, mainly related to religion (2). The COVID-19 vaccination received approval from the FDA in May 2021 (3). Children are considered more susceptible to COVID-19 than adults. However, several barriers to childhood vaccination programs persist, such as the parents’ assumption that children face lower risk of severe COVID-19 and are asymptomatic carriers (4). Another study mentioned that doubts about efficacy, concern about vaccine content, limited information on the vaccine from physicians, and fears regarding safety are factors contributing to parents’ refusal of childhood vaccination (5).

A previous study in Indonesia described parents’ concern to vaccinate their daughters with the HPV vaccine; this refusal was attributed to misinformation about the vaccine and its side effects, or a lack of sufficient information on the vaccine (6). This situation is similar to the context of the COVID-19 pandemic: according to a previous study, parents’ intention to vaccinate their children only reached 69%. Several predictors of parents’ refusal to vaccinate their children were concerns over vaccine side effects and unknown vaccine efficacy (7). The fact that 80% of Indonesian citizens are Muslims and the important role of neighborhood and/or religious leaders may also influence parents’ intention or refusal to vaccinate their children.

One theoretical framework to describe the process linking individual factors and a specific health behavior is the Health Belief Model (HBM). This model identifies six core components that may affect the likelihood of an individual performing a protective action related to health: (i) perceived susceptibility (assessment of the risk of acquiring a condition); (ii) perceived severity (assessment of the seriousness of the consequences of a condition if it is acquired); (iii) perceived benefits (assessment of the positive consequences of adopting a health behavior); (iv) perceived barriers (assessment of the influences that discourage adoption of a health behavior); (v) self-efficacy (the individual’s assessment of his/her ability to successfully adopt a health behavior); and (vi) cue to action (external influences that promote the health behavior) (8, 9). The HBM has also been applied to the analysis of behaviors related to COVID-19, including various COVID-19-preventive behaviors (10–12). Based on the previous studies, there are some gaps, such are: Cultural and Religious Specificity which the impact of religion on vaccine hesitancy across diverse religious groups or in non-Muslim contexts; Regional Diversity which many studies focus on specific regions, like Malaysia and Indonesia, but lack broader Southeast Asian or global comparisons; Age and Vaccine Type Specificity which HPV and COVID-19 vaccine hesitancy are the main focal points, leaving gaps in other vaccines or age groups; Broader Socioeconomic and Misinformation Influence which some studies lack an exploration of the impact of misinformation or economic constraints, which are significant factors in vaccine hesitancy. Addressing these gaps could involve a comprehensive study that examines vaccine hesitancy across various religious, cultural, and socioeconomic contexts, with a focus on both COVID-19 and other vaccines.

Developing the guideline for in depth interview is very important. An interview guide is crucial for enhancing the consistency, depth, and relevance of data collected, making it a cornerstone of reliable and effective qualitative research. By developing the in-depth interview guideline, we prepared the well-prepared questions and prompts ensure that the interview covers all aspects of the research topic. Although structured, a well-designed guide is also flexible, providing prompts that allow the interviewer to explore unexpected or emerging themes. This adaptability is critical in qualitative research, as it allows the interview to uncover insights that may not have been initially considered. Clear, open-ended questions in the guide encourage participants to provide detailed responses, which is essential for qualitative analysis. The guide also includes probing questions to prompt participants to expand on or clarify their answers, ensuring a richer understanding of the topic (13).

Indonesia’s COVID-19 vaccination program for children faces both potential benefits and barriers influenced by parents’ beliefs. Studying these factors through the HBM domains (Perceived Benefits and Perceived Barriers) allows public health officials to design interventions directly targeting parental attitudes toward vaccination. This study aims to develop guidance for in-depth interviews for a future qualitative study based on a cross-sectional quantitative study on parents with school-age children. The topic of the aforementioned qualitative study is the HBM of parents’ intention to vaccinate their children with the COVID-19 vaccine.

## 2 Materials and methods

### 2.1 Study design and sampling method

This study adopted a cross-sectional design. The study population was parents with 6–11-year-old children (COVID-19-vaccinated or not) in the central region of Java province. The subjects of this study were parents in the population criteria. We recruited the parents who had children with COVID-19 vaccinated, because this situation allowed researchers to uncover the factors that solidified their decision, providing a fuller understanding of both barriers and enablers within the HBM framework. This understanding can be instrumental in designing targeted interventions to increase vaccination rates among hesitant populations. The researchers bypass potential biases associated with hypothetical intention (e.g., stating an intention but never following through). This offers a clearer view of the factors that genuinely contribute to translating intention into behavior, as opposed to what participants think they would do. Some parents may have been hesitant at first, providing an opportunity to explore how their beliefs, perceptions, and attitudes evolved. This retrospective insight can identify critical turning points in decision-making that may be key to designing targeted interventions for hesitant parents.

The data were collected from 6th July to 10th August 2024 by filling in an online questionnaire.

### 2.2 Data collection process and measures

The questionnaire used in this study was adopted from Almalki et al. (14) and was translated into Bahasa Indonesia. This questionnaire has been validated and passed the reliability test. The content of the questionnaire focuses on parents’ intention to vaccinate their children and five domains of HBMs, namely: perceived susceptibility (3 items), perceived severity (3 items), perceived benefits (2 items), perceived barriers (4 items), and cues to action (2 items). The questionnaire used the Likert Scale, with possible response options as follows: 1 (strongly disagree), 2 (disagree), 3 (agree), and 4 (strongly agree).

Sample size was calculated using formulation in OpenEpi,^1^ with the following equation:

$n=\left[{\mathrm{DEFF}}^{*}\mathrm{Np}\left(1-p\right)\right]/\left[d2/Z21-\alpha /2^{*}\left(N-1\right)+p^{*}\left(1-p\right)\right]$

Sample sizes were provided for 95% confidence levels, with a statistical power of 80%.

### 2.3 Statistical analyses

The data were descriptively analyzed. The association between parents’ intention to vaccinate and domains of the HBM was assessed using Person correlation and linear regression analyses.

### 2.4 Ethics and consent

This study was approved by the Ethics Committee of University Muhammadiyah Gombong, under Approval Number 249.6/II.3.AU/F/KEPK/VIII/2023.

## 3 Results

**Figure 1:**
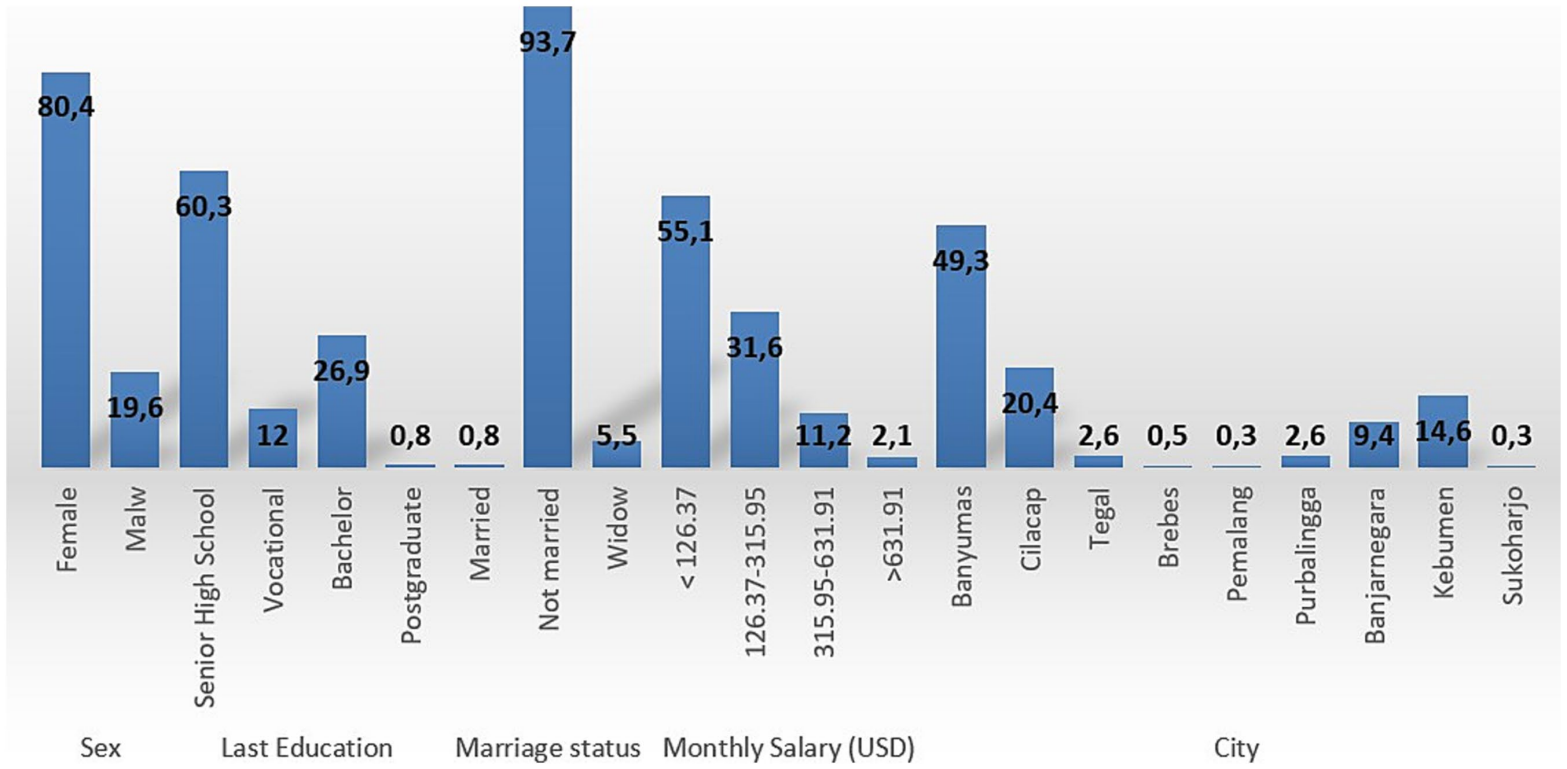
Description of respondents’ characteristics (%).

We recruited 382 subjects from the Central Java province. Figure 1 and Table 1 presents the respondents’ characteristics. Most of the respondents were female (80.4%), with the highest education level being senior high school (60.3%). Marital status was predominantly married (93.7%), and most of the respondents earned a monthly wage of below 126. Interestingly, most of the subjects mentioned that none of their family members (68.4%) or their children (95.3%) had been infected to date. Most respondents indicated that their children had received two doses of the COVID-19 vaccine. Parents obtained information from social media (48.8%) and their commitment to provide the influenza vaccine to their children was high (55.9%). Table 2 describes the significant association among parents’ intention to vaccinate their children and perceived susceptibility, perceived severity, perceived benefits, perceived barriers, and cues to action (*p* < 0.05). These associations indicate that parents’ decision to vaccinate their children is influenced by concerns relating to these five dimensions. These associations were adjusted to subjects’ characteristics, described in Table 3. There was no significant association between parents’ intention to vaccinate their children and their characteristics (Table 3). Owing to the objective of this study (i.e., to prepare guidance for developing in-depth interviews for a qualitative study on this topic) we continued our analysis with linear regression (Table 4). We found that the most significant association was between parents’ intention to vaccinate and perceived benefits and perceived barriers (*p* < 0.001). Guidance for developing the in-depth interview in a qualitative study was developed based on the present study’s findings. Table 5 lists in-depth interview points for the qualitative study.

**Table 1:** Respondents’ characteristics.

Characteristics	Mean (SD)	
Age	37.34 ± 7.394	
Characteristics	Number (N)	Percentage (%)
Have you or adults living with you been infected with SASR-CoV-2 since the beginning of the pandemic?		
Yes, I am or other family member is currently infected	105	27.4
Yes, some of us have been but are now healthy	16	4.2
No, we have not been infected by the virus	226	68.4
Have any of your children been infected with SARS-CoV-2?		
No, they have not been infected	365	95.3
Yes, they have previously been infected, but are recently healthy	10	2.6
Yes, one or more are currently infected	8	2.1
Are you vaccinated, and how many dosages did you receive?		
Yes, one dosage	17	4.4
Yes, two dosages or more	357	93.2
No, I am not	8	2.1
No, because I am exempted	1	0.3
If you have children over 12 years old and under 18 years old, have they been vaccinated at least once for COVID-19?		
Yes, they have been vaccinated once	43	11.2
Yes, they have been vaccinated twice	167	43.6
I do not have children over 12 years old	156	40.7
No, they haven’t been vaccinated	13	3.4
No, because they are exempted	4	1.0
My main source of information on COVID-19 vaccine is		
Social medial	187	48.8
Television	110	28.7
Friends/family	43	11.2
Others	43	11.2
How do you rate your children’s health in general (aged 5-11 years old)?		
Very good	125	32.6
Good	243	63.4
Average	15	3.9
Are any of your children (5–11 years old) suffering from chronic illness that require them regular medications?		
Yes	16	4.2
No	367	95.8
I commit to giving my children (5–11 years) the annual seasonal influenza vaccine		
Yes	169	44.1
No	214	55.9

**Table 2:** Association among parents’ intention to vaccinate their children and domains of health belief model.

	Parents’ intention (Number of subjects)				*p*-value
	Definitely Yes	Maybe Yes	Maybe Not	Definitely Not	
Perceived susceptibility					
Strongly agree	1	1	0	0	0.028^*^
Agree	42	34	5	11	
Disagree	74	98	21	30	
Strongly disagree	22	22	11	11	
Perceived severity					
Strongly agree	11	10	1	1	<0.001^*^
Agree	99	113	21	25	
Disagree	26	28	14	20	
Strongly disagree	3	4	1	6	
Perceived benefits					
Strongly agree	41	13	2	1	<0.001*
Agree	91	136	25	27	
Disagree	4	6	8	20	
Strongly disagree	3	0	2	4	
Perceived barriers					
Strongly agree	3	4	5	10	<0.001*
Agree	74	116	24	37	
Disagree	60	35	7	3	
Strongly disagree	2	0	1	2	
Cues to action					
Strongly agree	15	4	3	4	<0.001*
Agree	121	144	27	34	
Disagree	0	7	7	12	
Strongly disagree	1	0	0	2	

* Significant association.

**Table 3:** Description of parents’ intention to vaccinate their children based on their characteristics.

	Parents’ intention (Number of subjects)				*p*-value
	Definitely Yes	Maybe Yes	Maybe Not	Definitely Not	
Sex					
Male	31	27	6	11	0.664
Female	108	128	31	41	
Marriage status					
Married	131	148	34	46	0.078
Widow	8	6	2	5	
Not married	0	1	1	1	
Last education					
Up to Senior high school	81	95	20	35	0.676
Vocational	18	21	4	3	
Bachelor	38	39	13	13	
Postgraduate	2	0	0	1	
Monthly salary (IDR)					
<2.000.000	74	87	18	32	0.313
2.000.000–5.000.000	41	52	16	12	
5.000.000–10.000.000	19	14	52	7	
>10.000.000	5	2	41	1	
	Definitely Yes (Mean ± SD)	Maybe Yes (Mean ± SD)	Maybe Not (Mean ± SD)	Definitely Not (Mean ± SD)	
Age	37.81 ± 7.100	37.12 ± 7.643	36.24 ± 8.060	37.51 ± 7.001	0.678

**Table 4:** Linear regression results of the parents’ intention to vaccine COVID-19.

Independent variable	*B*	Beta	*t*	Sig
(Constant)	1.796		4.293	0.000
Perceived susceptibility	0.025	0.045	0.925	0.356
Perceived severity	0.532	0.094	1.677	0.094
Perceived benefits	0.301	0.343	6.346	0.000
Perceived barriers	−0.157	−0.313	−7.190	0.000
Cues to action	0.002	0.002	0.037	0.970

**Table 5:** List of questions in the in-depth interview of qualitative study.

Dimensions	Questions
Introduction	How was your experience during the COVID-19 pandemic?
	Have any of your family members been infected with COVID-19?
	Who were they and when? Has your child ever been diagnosed with COVID-19?
	IF YES
Perceived severity	What happened to your child when he or she got COVID-19? What were the symptoms? What was the impact on his physical/emotional/social/school?
	When your child experienced COVID-19, what was the impact on your family and people around you? (for example, parents cannot work because they have to take care of them, siblings have to move temporarily, etc.)
	Are there any long-term impacts experienced by your children as a result of COVID-19 that they have experienced (long-Covid)?
	Was the impact of COVID-19 on children different from the impact on adults? (the same/worse/lighter, please explain?)
	IF YES
	What do you know about the symptoms of COVID-19 in children? What is the impact on the physical/emotional/social/school of children affected by COVID-19?
	What do you know about the impact of a child diagnosed with COVID-19 on the family and people around them? (for example, parents cannot work because they have to take care of them, siblings have to move temporarily, etc.)
	What do you know about the long-term impact experienced by children affected by COVID-19 (long-Covid)?
	Is the impact of COVID-19 on children different from the impact on adults? (the same/worse/lighter, please explain?)
Perceived susceptibility	In your opinion, how vulnerable are your children to COVID-19?
	What is the reason? Compared to adults, who is more vulnerable?
	Are you worried that your child will get COVID-19?
	Are you taking special measures to protect your children from COVID-19? If so, what are they?
Perceived benefits	Has your child been vaccinated against COVID-19? How many times? When was that?
	What do you know in general about vaccines? What are the benefits of vaccines?
	What do you think are the main benefits of the COVID-19 vaccine for children?
	Has the COVID-19 vaccine that your child has received had a good impact on him so far? If so, what are the outcome (e.g., mild symptoms, no longer getting a second covid, etc)? What is the reason if there are no outcomes?
	Do you see vaccines as an effective way to protect your child from COVID-19?
Perceived barriers	Do you have any specific concerns about COVID-19 vaccines for children?
	What are the main considerations for you in deciding whether or not to vaccinate your child related to COVID-19?
	Are there certain events or information that affect your decision regarding the administration of the COVID-19 vaccine for children? Suppose there is influence from family or neighbors
	What are the obstacles or obstacles that you feel in deciding to give the COVID-19 vaccine to your child?
	If your child has been vaccinated, what is the process? Are there any side effects experienced? or what symptoms occur after your child is vaccinated?
Cues to action	Where do you get information about COVID-19 vaccines for children? What form of information about COVID-19 and the COVID-19 vaccine is the easiest for you to accept and understand? (e.g., TV advertisements, flyers, lectures at ibvadah’s house, discussions with health workers, etc.)
	Regarding the administration of the COVID-19 vaccine to your children, who is the party that you trust their opinions/suggestions so that you will be willing to give vaccines to your children? (ex: government, doctors, pharmacists, religious experts, celebrities, etc.) Why are these people you trust?
Additional questions	What do you think about the government’s policy regarding COVID-19 vaccination for children?
	Do you think vaccination should be mandatory for children?
	If the COVID-19 vaccine is not mandatory, will you continue to give the COVID-19 vaccine for children?

## 4 Discussion

The present study aimed to develop guidance for in-depth interview for a future qualitative study based on a cross-sectional quantitative study of parents with school-age children. All parents reported that their decision on whether or not to vaccinate their children had been made taking into many considerations, including the mandatory procedures for continuing offline learning in schools. Our study finds that perceived benefit and perceived barriers were the two domains that significantly influenced parents’ intention to vaccinate their children. Our results are in line with those of several previous study. The study reported that parents are concerned about the dangers of COVID-19 infection and the benefit of the vaccine. Thus, perceived severity and benefits were found to be dominant in that study (15). In our study, there was no significant association between parent gender and the intention to vaccinate their children. A previous study mentioned that fathers had higher willingness to vaccinate their children, because of the riskier behaviors. Men are available in working area or outside of the house situation, which could be more possibility to be exposed by the virus. Mothers also had to make a decision about their children health, because they have more time spent daily (16, 17). Our study shows that parents’ acceptance of vaccinating their children is high. However, another study reported that parents’ acceptance of vaccination is very low, and that factor associated with this lack of acceptance included access to information, trust, and community norms. To address these concerns, education programs should be made available to enable the community to obtain accurate information (3). The role of neighborhood leaders or religion/spiritual leaders in imparting suitable information about the importance of vaccination in children is also crucial.

A systematic review and meta-analysis about parents and guardians’ willingness to vaccinate their children indicated that perceived susceptibility, perceived severity, and perceived benefits had significant associations with parents’ and guardians’ willingness to vaccinate their children. Parents or guardians’ willingness to vaccinate themselves was an important predictor of their willingness to vaccinate their children (18). A previous study in Saudi Arabia reported that all HBM domains were significantly associated with parents’ intention to vaccinate their children. The most strongly associated domains were perceived benefit and perceived barriers (14). This result is in line with our finding, given the background of the two countries is similar: Saudi Arabia is a well-known Muslim country, and 80% of Indonesian citizens are Muslims. We included religion as a consideration because religious leaders have a significant role in promoting vaccination programs through collaborating the science and religion. However, some religious leaders have been reported to spread misinformation about the side effects of vaccination (19). A previous study mentioned that information from religious leaders can influence Muslims’ attitudes and decisions regarding vaccination. Thus, it is important to involve religious leaders in immunization programs, as Muslims require reassurance that vaccines follow the Shariah rules (2).

The differences between our study and previous works is that our study adopted a cross-sectional design with a questionnaire to assess the association between parents’ intentions to vaccinate their children, using HBM. We further developed guidance for in-depth interviews to find out more about parents’ intentions to vaccinate their children and the related factors. As mentioned before, a significant association was observed for perceived benefits and barriers. Thus, we emphasized questions related to benefits and barriers in the interview guidelines. However, we planned to start the interview with questions on perceived severity for more prolonged engagement. We expect that parents will respond positively to the interview process if asked to describe their experiences during the COVID-19 pandemic: based on our experiences, it is difficult to find prospective qualitative respondents. They might refuse to participate because of their concerns regarding vaccination. Thus, strategies aimed at prolonged engagement were applied at the start of the interview. The first qualitative study about the COVID-19 vaccine also suggested this method (20).

In our guidance for in-depth interviews, the content on perceived severity included questions about the symptoms experienced by children due to COVID-19 infection, the short term and long-term impact, and the impact on children relative to adults. Questions about parents’ knowledge about COVID-19 symptoms, its impact, and the severity of impact were also explored in this domain. The content on perceived severity explored the level of severity for children, the concerns of parents about COVID-19 infection, and the steps taken by parents to protect their children. The perceived benefits explored the advantages of COVID-19 vaccination. The content on perceived barriers included concerns of parents, information influencing the decision to receive the vaccination, the procedure vaccination and the effect after vaccination. The domain of cues of action explored sources of information about vaccination and people trusted by the parents. The additional information content included questions about the government policy, especially related to the mandatory aspect of the COVID-19 vaccination for children. We arranged these questions based on our experience during the quantitative study as well as previous reports (14, 15, 17, 20). Regarding the development of the guidance for in-depth interviews, we agree that more explicit details would benefit other researchers. While this was largely an exploratory process, our experiences allowed us to refine our questions, identify common themes, and avoid potential pitfalls in future qualitative interviews. To enhance transparency and reproducibility, we will provide a detailed outline of how our quantitative findings informed the qualitative phase, including specific challenges, adaptations made, and lessons learned.

Since parents’ perceptions of vaccine benefits and barriers are likely influenced by cultural, religious, and socioeconomic contexts, findings may not automatically transfer across diverse populations. Generalizability could be improved by detailing these factors within the study, allowing others to judge the applicability of the results to their own populations or settings. The Health Belief Model (HBM) provides a structured framework (e.g., perceived benefits, perceived barriers, cues to action), making it easier to adapt findings to other studies. While specific findings on barriers and benefits may vary, the core HBM domains offer a transferable structure. Emphasizing these core domains can aid in the study’s adaptation to different regions, cultures, or vaccines. The in-depth interview guidelines developed in the study can be made more widely applicable by including flexible prompts that allow researchers to explore culturally specific or context-specific factors related to vaccine hesitancy. By doing so, the guidelines could serve as a useful tool for conducting qualitative research on vaccine intention in different settings. We also still used some questions in the interview guideline which is related to the HBM, because, the parents’ intention to do the vaccination for their children could influence by complex constructs.

The strength of our study was that we used a quantitative study with large number of respondents and experience of the researcher during the quantitative study to develop a guidance of an in-depth interview of the qualitative study. The limitation of this study is that, we cannot include religion aspect in the guidance, because we will select the subject randomly, not based on religion. Thus, the exploration about religion could be mentioned by the subjects, if they have experience about the influence of religion in decision making. The role of religion leader, is one of the important factors which influenced the decision maker about the use of drug, including vaccine. In Indonesia, there is a Muslim Board, namely Majelis Ulama Indonesia (MUI) which has responsibility in the recognition of halal products, including food and drinks, cosmetics, drugs and vaccine. Most of the muslim’ people will comply with the decision of MUI regarding the use of some products. MUI was formed by the government and the main role is conducting the comprehensive review and analysis about the halal of product. Thus, it is important to add the religion leaders as part of the interview.

In the future, it is important to implement some programs, such as health promotion and cadres’ education to increase the community awareness about the vaccination. The interview guideline also can be implemented in the intervention study to explore the HBM before and after the intervention.

## 5 Conclusion

The significant association observed between parents’ intention in some cities of Central of Java, to vaccinate their children and perceived benefits and perceived barriers yielded guidance for in-depth interviews in the qualitative study. We belief that the interview guideline can be implemented in Central of java area, because we selected respondents from big cities in Central of java, randomly. The Health Belief Model should be further explored in Indonesia because several external factors may influence parents’ intention to vaccinate their children.

## Data Availability

The raw data supporting the conclusions of this article will be made available by the authors, without undue reservation.

## Footnotes

^1^ www.OpenEpi.com

## References

[1] Tanne JH. COVID-19: FDA panel votes to approve Pfizer BioNTech vaccine. BMJ. (2020) 371:m4799. doi: 10.1136/bmj.m4799, PMID: 33310748

[2] Alsuwaidi AR Hammad HAAK Elbarazi I Sheek-Hussein M. Vaccine hesitancy within the Muslim community: Islamic faith and public health perspectives. Hum Vaccin Immunother. (2023) 19:2190716. doi: 10.1080/21645515.2023.2190716, PMID: 36914409 PMC10038058

[3] Hon Snir S Teitler Regev S. I have decided about my COVID-19 vaccine, what about my child? Hum Vaccin Immunother. (2022) 18:2129929. doi: 10.1080/21645515.2022.2129929, PMID: 36315873 PMC9746399

[4] Galanis P Vraka I Siskou O Konstantakopoulou O Katsiroumpa A Kaitelidou D. Willingness, refusal and influential factors of parents to vaccinate their children against the COVID-19: a systematic review and meta-analysis. Prev Med. (2022) 157:106994. doi: 10.1016/j.ypmed.2022.106994, PMID: 35183597 PMC8861629

[5] Ng DLC Gan GG Chai CS Anuar NAB Sindeh W Chua WJ. The willingness of parents to vaccinate their children younger than 12 years against COVID-19: a cross-sectional study in Malaysia. BMC Public Health. (2022) 22:1265. doi: 10.1186/s12889-022-13682-z, PMID: 35768789 PMC9241237

[6] Wijayanti KE Schutze H Mac Phail C Ivers R. Exploring parents’ decisions regarding HPV vaccination for their daughters in Jakarta, Indonesia: a qualitative study. Asian Pac J Cancer Prev. (2023) 24:3993–8. doi: 10.31557/APJCP.2023.24.11.3993, PMID: 38019260 PMC10772741

[7] Santi T Hegar B Munasir Z Prayitno A Werdhani RA Bandar INS. Factors associated with parental intention to vaccinate their preschool children against COVID-19: a cross-sectional survey in urban area of Jakarta, Indonesia. Clin Exp Vaccine Res. (2023) 12:240–8. doi: 10.7774/cevr.2023.12.3.240, PMID: 37599811 PMC10435772

[8] Saunders GH Frederick MT Silverman SC Nielsen C Laplante-Lévesque A. Description of adults seeking hearing help for the first time according to two health behavior change approaches: transtheoretical model (stages of change) and health belief model. Ear Hear. (2016) 37:324–33. doi: 10.1097/AUD.0000000000000268, PMID: 26765286

[9] Rosenstock IM. The Health Belief Model and Personal Health Behavior/charles B In: Health education monographs, vol. 2: ed. Green L. W. Slack, Inc (1974). 328–35. http://www.jstor.org/stable/45240623

[10] Alagili DE Bamashmous M. The health belief model as an explanatory framework for COVID-19 prevention practices. J Infect Public Health. (2021) 14:1398–403. doi: 10.1016/j.jiph.2021.08.024, PMID: 34462221 PMC8386094

[11] Guidry JPD O’Donnell NH Austin LL Coman IA Adams J Perrin PB. Stay socially distant and wash your hands: using the health belief model to determine intent for COVID-19 preventive behaviors at the beginning of the pandemic. Health Educ Behav. (2021) 48:424–33. doi: 10.1177/10901981211019920, PMID: 34185596

[12] Tadesse T Alemu T Amogne G Endazenaw G Mamo E. Predictors of coronavirus disease 2019 (COVID-19) prevention practices using health belief model among employees in Addis Ababa, Ethiopia, 2020. Infect Drug Resist. (2020) 13:3751–61. doi: 10.2147/IDR.S275933, PMID: 33122922 PMC7588498

[13] Hemming J Lordly D Glanville NT Corby L Thirsk J. Developing an interview guide to evaluate practice-based evidence in nutrition: use of the Delphi technique. Can J Diet Pract Res Publ Dietitians. (2011) 72:186–90. doi: 10.3148/72.4.2011.186 22146118

[14] Almalki OS Alfayez OM Al Yami MS Asiri YA Almohammed OA. Parents’ hesitancy to vaccinate their 5-11-year-old children against COVID-19 in Saudi Arabia: predictors from the health belief model. Front Public Health. (2022) 10:842862. doi: 10.3389/fpubh.2022.842862, PMID: 35433579 PMC9005777

[15] Rajeh MT Farsi DJ Farsi NJ Mosli HH Mosli MH. Are parents’ willing to vaccinate their children against COVID-19? A qualitative study based on the health belief model. Hum Vaccin Immunother. (2023) 19:2177068. doi: 10.1080/21645515.2023.2177068, PMID: 36755490 PMC10054307

[16] Teasdale CA Borrell LN Kimball S Rinke ML Rane M Fleary SA. Plans to vaccinate children for coronavirus disease 2019: a survey of United States parents. J Pediatr. (2021) 237:292–7. doi: 10.1016/j.jpeds.2021.07.021, PMID: 34284035 PMC8286233

[17] Wan X Huang H Shang J Xie Z Jia R Lu G. Willingness and influential factors of parents of 3-6-year-old children to vaccinate their children with the COVID-19 vaccine in China. Hum Vaccin Immunother. (2021) 17:3969–74. doi: 10.1080/21645515.2021.1955606, PMID: 34344258 PMC8828112

[18] Chen F He Y Shi Y. Parents’ and guardians’ willingness to vaccinate their children against COVID-19: a systematic review and meta-analysis. Vaccine. (2022) 10:2–179. doi: 10.3390/vaccines10020179, PMID: 35214638 PMC8880569

[19] Galang JRF. Science and religion for COVID-19 vaccine promotion. J Public Health. (2021) 43:e513–4. doi: 10.1093/pubmed/fdab128, PMID: 33866364 PMC8083241

[20] Ogueji IA Okoloba MM. Underlying factors in the willingness to receive and barriers to receiving the COVID-19 vaccine among residents in the UK and Nigeria: a qualitative study. Curr Psychol. (2022) 42:13455–66. doi: 10.1007/s12144-021-02498-6, PMID: 35043039 PMC8758235

